# Effects on Right Ventricular Function One Year after COVID-19-Related Pulmonary Embolism

**DOI:** 10.3390/jcm12113611

**Published:** 2023-05-23

**Authors:** Federica Ilardi, Mario Crisci, Cecilia Calabrese, Anna Scognamiglio, Fortunato Arenga, Rachele Manzo, Domenica F. Mariniello, Valentino Allocca, Anna Annunziata, Antonello D’Andrea, Raffaele Merenda, Vittorio Monda, Giovanni Esposito, Giuseppe Fiorentino

**Affiliations:** 1 Department of Advanced Biomedical Sciences, Federico II University Hospital, Via S. Pansini, 5, 80131 Naples, Italy; 2 Department of Cardiology, Division of Interventional Cardiology, Monaldi Hospital, 80131 Naples, Italy; 3 Department of Translational Medical Sciences, University of Campania “Luigi Vanvitelli”, Monaldi Hospital, 80131 Naples, Italy; 4 Department of Intensive Care, A.O.R.N dei Colli, Monaldi Hospital, 80131 Naples, Italy; 5 Unit of Cardiology and Intensive Coronary Care, “Umberto I” Hospital, 84014 Nocera Inferiore, Italy

**Keywords:** COVID-19, pulmonary embolism, right-ventricle global longitudinal strain, right-ventricle free wall longitudinal strain, right-ventricle dysfunction

## Abstract

The aim of this study was to investigate the presence of subclinical cardiac dysfunction in recovered coronavirus disease 2019 (COVID-19) patients, who were stratified according to a previous diagnosis of pulmonary embolism (PE) as a complication of COVID-19 pneumonia. Out of 68 patients with SARS-CoV-2 pneumonia followed up for one year, 44 patients (mean age 58.4 ± 13.3, 70% males) without known cardiopulmonary disease were divided in two groups (PE+ and PE−, each comprising 22 patients) and underwent clinical and transthoracic echocardiographic examination, including right-ventricle global longitudinal strain (RV-GLS), and RV free wall longitudinal strain (RV-FWLS). While no significant differences were found in the left- or right-heart chambers’ dimensions between the two study groups, the PE+ patients showed a significant reduction in RV-GLS (−16.4 ± 2.9 vs. −21.6 ± 4.3%, *p* < 0.001) and RV-FWLS (−18.9 ± 4 vs. −24.6 ± 5.12%, *p* < 0.001) values compared to the PE- patients. According to the ROC-curve analysis, RV-FWLS < 21% was the best cut-off with which to predict PE diagnosis in patients after SARS-CoV-2 pneumonia (sensitivity 74%, specificity 89%, area under the curve = 0.819, *p* < 0.001). According to the multivariate logistic regression model, RV-FWLS < 21% was independently associated with PE (HR 34.96, 95% CI:3.24–377.09, *p* = 0.003) and obesity (HR 10.34, 95% CI:1.05–101.68, *p* = 0.045). In conclusion, in recovered COVID-19 patients with a history of PE+, there is a persistence of subclinical RV dysfunction one year after the acute phase of the disease, detectable by a significant impairment in RV-GLS and RV-FWLS. A reduction in RV-FWLS of lower than 21% is independently associated with COVID-related PE.

## 1. Introduction

Coronavirus disease-2019 (COVID-19) is a systemic viral infection caused by severe acute respiratory syndrome coronavirus 2 (SARS-CoV-2), with a predominantly respiratory clinical presentation, and associated with multi-organ complications. Cardiac involvement has been frequently described, both as the effect of direct infection and because of increased pulmonary resistance due to acute respiratory distress syndrome (ARDS) and pulmonary embolism (PE) [1,2]. Pulmonary embolism imposes a pressure overload on the right-heart chambers, leading to progressive right ventricle (RV) dilation and dysfunction [3]. Echocardiographic evidence of adverse RV remodeling in the acute phase of COVID-19 pneumonia has been extensively described and associated with early mortality, independently of clinical and biomarker risk stratification [4]. In this context, two-dimensional (2D) speckle-tracking derived RV longitudinal strain was shown to be more effective than conventional echocardiographic parameters in detecting early and subclinical signs of RV dysfunction, as well as providing additional predictive value [5]. However, to date, little is known about the persistence of myocardial injury in recovered COVID-19 patients, with or without a previous diagnosis of PE. The aim of the present study is to evaluate the presence of echocardiographic signs of myocardial dysfunction in COVID-19-pneumonia survivors at one year of follow-up, according to their positive/negative clinical history of PE.

## 2. Materials and Methods

### 2.1. Patient Population and Data Collection

Patients hospitalized between March 2020 and March 2021 with a diagnosis of SARS-CoV-2 pneumonia, confirmed by real-time reverse-transcription polymerase chain reaction on naso-pharyngeal swab and high-resolution computed tomography (HRCT) of the lung, either complicated with PE or not, and recovered from COVID-19, who agreed to be followed up in the outpatient clinic, were screened. Exclusion criteria were previous history of coronary artery disease, arrhythmia, valvular heart disease, chronic obstructive pulmonary disease, bronchial asthma, and obstructive sleep apnea. Forty-six out of sixty-eight initially screened patients met the inclusion criteria and were considered eligible for the present study. All enrolled patients had either severe COVID-19 pneumonia (in presence of fever, cough, dyspnea, fast breathing, and one of respiratory rate > 30 breaths/min, severe respiratory distress, or peripheral capillary oxygen saturation [SpO2] < 90% in room air) or critical with mild ARDS (P/F between 200 and 300 mmHg, with either PEEP or cPAP ≥ 5 cm H_2_O) [6]. Degree of pulmonary involvement in each patient was evaluated through CT scan using an overall lung “total severity score”: no involvement corresponded to a lobe score of 0, minimal involvement to a lobe score of 1, mild involvement to a lobe score of 2, moderate involvement to a lobe score of 3, and severe involvement to a lobe score of 4. The total severity score was reached by summing the five lobe scores (range of possible scores, 0–20) [7].

**Figure 1:**
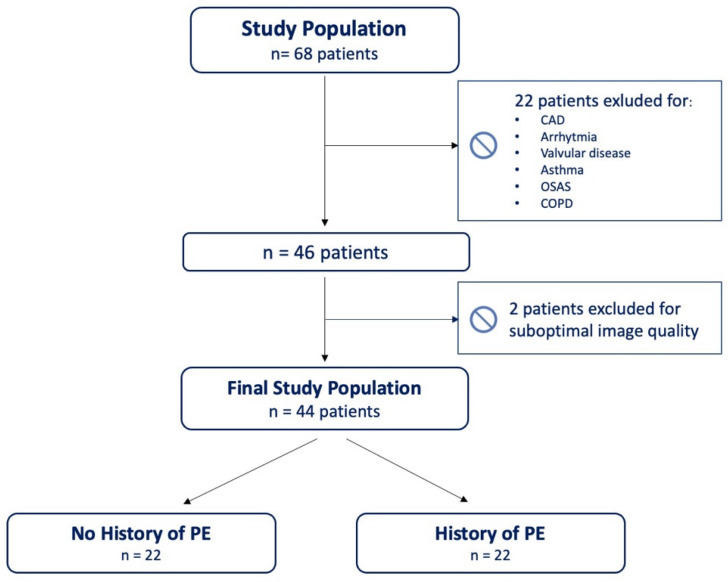
Study design. CAD = coronary artery disease; COPD = chronic obstructive pulmonary disease; OSAS = obstructive sleep apnea syndrome; PE = pulmonary embolism.

Patients included in the study were divided into two subgroups based on their positive/negative clinical history of PE as a complication of COVID-19 pneumonia, i.e., PE+ and PE−, respectively. The PE diagnoses were confirmed by CT pulmonary angiography. Those who experienced an incident thromboembolic event were managed with therapeutic doses of anticoagulant therapy. After discharge, PE+ patients were treated with direct oral anticoagulants (DOACs) for at least 6 months. All patients underwent a 12-month visit involving clinical and physical examination, including SpO2 measured by pulse oximetry on the index finger, electrocardiogram, and a complete echocardiographic examination. Two patients were excluded from the final analysis due to suboptimal image quality. The study design is summarized in Figure 1.

### 2.2. Echocardiographic Measurements

Transthoracic echocardiograms were performed using a Vivid ultrasound (E9) System (GE Healthcare, Horten, Norway) and stored on a dedicated workstation for offline analysis (EchoPAC, GE Healthcare, Chicago, IL, USA, Version 203). For each echocardiographic measurement, at least two cardiac cycles were averaged. Conventional echocardiographic measurements were performed in accordance with the guidelines [8,9]. Right atrial and RV size were determined from the apical 4-chamber RV-focused view. Tricuspid annular plane systolic excursion (TAPSE) was measured as the systolic displacement of the tricuspid lateral annulus, recorded on M-mode imaging. The RV fractional area change (RVFAC) was calculated as: (RV end-diastolic area − RV end-systolic area)/end-diastolic area × 100%. Tricuspid lateral annular systolic velocity (S′) was assessed using tissue Doppler imaging from the apical 4-chamber view. Pulmonary artery systolic pressure (PASP) was assessed from the peak velocity of the tricuspid regurgitation jet, using the modified Bernoulli equation plus right atrial pressure evaluated based on the inferior vena cava size and collapsibility [10]. Distal RV outflow tract (RVOT) diameter was measured in a parasternal short-axis view. In the same view, RVOT acceleration time was obtained from the RVOT pulsed-wave Doppler profile.

Strain analysis, based on the speckle-tracking approach, was measured by an experienced cardiologist, as previously described [11,12], and expressed as an absolute value. Image acquisition for the measurement of left ventricle (LV) global longitudinal strain (GLS) was performed in apical long-axis, 4- and 2-chamber views (frame rate 50–90 frame/s). The RV longitudinal strain was obtained from the apical 4-chamber RV-focused view [13]. After tracing the RV endocardial border, the region of interest was automatically generated; next, manual corrections were performed to fit the RV myocardial wall thickness. The RV free wall and interventricular septum were each automatically divided into three segments (basal, mid, and apical). The RV-GLS was calculated as the average of the six segments; RV free wall longitudinal strain (RV-FWLS) was calculated as the average of the three segments. If it was not feasible to track one or more segments, the patient was excluded.

### 2.3. Statistical Analysis

Normality of the distribution of continuous variables was tested by the Kolmogorov–Smirnov test. All data were expressed as mean ± standard deviation (SD) or median (interquartile range) as appropriate for continuous variables or percentages of individuals for categorical variables. Differences between groups were analyzed for statistical significance with the unpaired *t*-test for normally distributed continuous variables, the Mann–Whitney U test for non-normally distributed continuous variables, and chi-square test for categorical variables.

Receiver-operator characteristics (ROC) curve was generated and the Youden’s J statistic was used to estimate the best cut-off value of RV-FWLS to predict the presence of PE. Multivariate logistic regression model was applied to determine the independent association with RV-FWLS < 21%. The selection of the variables for the multivariable analysis was based on their significant association (*p*-value < 0.1) with RV-FWLS < 21% according to the univariate regression analysis. Parameters included in the multivariate analysis, with a stepwise backward-elimination method, were PE, obesity, hypertension, and RVOT acceleration time. Statistical analyses were performed using IBM-SPSS, version 23 (SPSS Inc., Chicago, IL, USA). A *p*-value < 0.05 was considered significant.

## 3. Results

Of the patients followed up for SARS-CoV-2 pneumonia, 44 fulfilled the inclusion criteria and were included in this analysis, of whom 22 PE+ and 22 PE−. The baseline demographic, clinical, and biological characteristics of the study cohort are presented in Table 1.

The population was predominantly male (70%, mean age 58.4 ± 13.3 years), hypertensive (66%), and obese (43%, mean BMI 30.3 ± 6.3 kg/m^2^). The two populations did not differ in terms of age, gender, or cardiovascular risk factors. The patients affected by COVID-19 complicated by PE+ had longer hospitalizations than those with PE− (30.2 ± 10.3 vs. 17.7 ± 11.6 days, *p* = 0.001), but mechanical ventilation and hemodynamic support were only needed in 9% of the cases. In the PE+ group, higher values of IL-6 (279 (159–544) vs. 50 (29–365), *p* = 0.017) and D-dimer (620 (293–2898) vs. 340 (88–1099), *p* = 0.025) were observed compared to the PE− group, with no significant differences in the levels of troponin and C-reactive protein. Moreover, a higher degree of pulmonary lobe involvement was observed in the PE+ group than in the PE− group (lung total severity score 10.8 ± 3.1 vs. 7.3 ± 3.9, *p* = 0.004).

At one-year follow-up, the comparison between the PE+ and PE− subgroups did not disclose any significant difference in clinical status (Supplemental Table S1).

The echocardiographic assessments performed one year after SARS-CoV-2 pneumonia showed that the patients with PE+ had comparable LV dimensions, global and longitudinal systolic function, and atrial volume with those of the patients with PE− (Table 2).

Conversely, the E/e′ values were significantly higher in the PE+ patients compared to the PE- patients (9.3 ± 2.5 vs. 7.0 ± 2.0, *p* = 0.015), although they were still normal. Despite no significant differences were found in right-chamber dimensions and RV systolic function between the two study groups, the PE+ patients had more significant reductions in RV-GLS (16.4 ± 2.9 vs. 21.6 ± 4.3%, *p* < 0.001) and RV-FWLS (18.9 ± 4 vs. 24.6 ± 5.12%, *p* < 0.001) compared to the PE− patients (Table 3).

**Figure 2:**
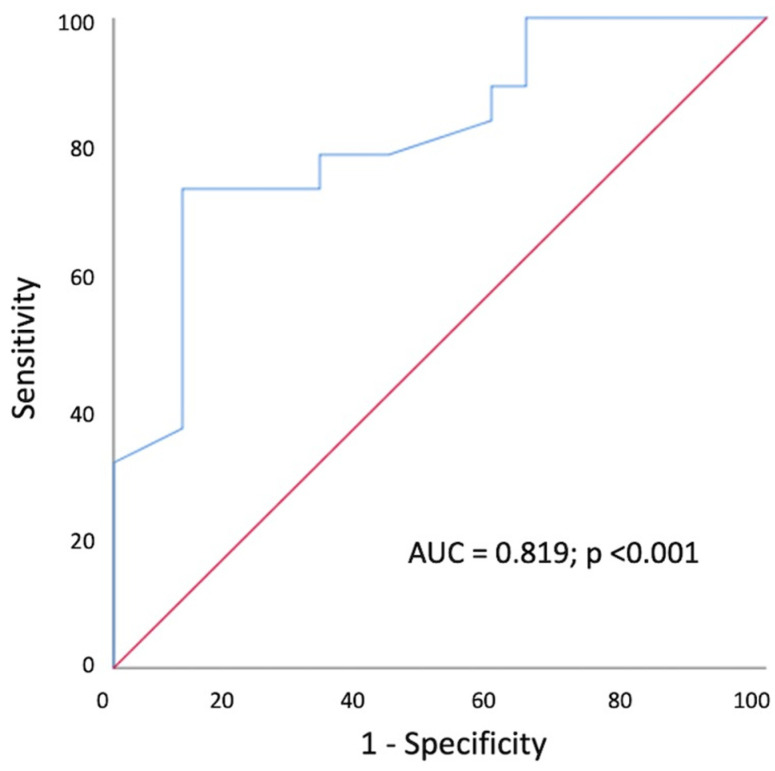
ROC curve of RV free wall longitudinal strain associated with pulmonary embolism in COVID-19 survivors.

Moreover, a trend towards an increase in tricuspid valve velocity (2.36 ± 0.32 vs. 2.15 ± 0.33 m/s, *p* = 0.073) and tricuspid regurgitation gradient (22.7 ± 5.7 vs. 18.8 ± 6 mmHg, *p* = 0.060) was observed in the PE+ group, together with a slight, insignificant reduction in RVOT acceleration time (95.9 ± 18.8 vs. 109.2 ± 26 msec, *p* = 0.07) (Table 3). According to the ROC-curve analysis (Figure 2) RV-FWLS < 21% was the best cut-off with which to predict PE diagnosis in patients after SARS-CoV-2 pneumonia (sensitivity 74%, specificity 89%, area under the curve = 0.819, *p* < 0.001).

According to the multivariate logistic regression model, after adjustment for RVOT acceleration time and hypertension, a value of RV-FWLS < 21% was independently associated with obesity (HR 10.34, 95% CI 1.05–101.68, *p* = 0.045) and PE+ (HR 34.96, 95% CI 3.24–377.09, *p* = 0.003) (Table 4).

## 4. Discussion

**Figure 3:**
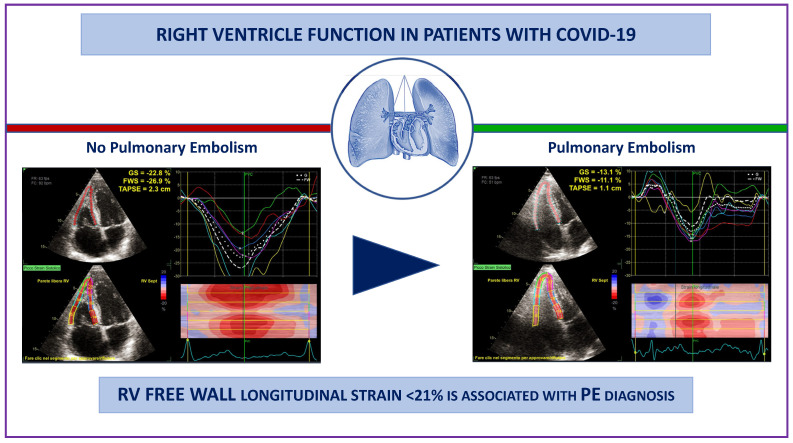
Analysis of RV TAPSE, global strain and free wall strain in representative cases of COVID-19 recovered patients, with (**left panel**) and without (**right panel**) previous diagnoses of pulmonary embolism.

In the current study, we evaluated the impact of SARS-CoV-2 pneumonia, either complicated by PE or not, on myocardial function at one-year follow-up. We showed that in the COVID-19 survivors with previous PE, RV-GLS and RV-FWLS were impaired compared to those without PE. Our findings suggest that RV-FWLS < 21% is strongly associated with previous PE in COVID-19 survivors (Figure 3).

Dysfunction in RV has emerged as a common feature of COVID-19, most significantly in patients with ARDS [14,15]. In this setting, indeed, the inflammatory and prothrombotic responses triggered by the viral infection encourage micro- and macrothromboses, which cause PE, as well as sustained vasoconstriction, leading to increases in pulmonary vascular resistance. All these modifications in the pulmonary vascular bed result in hydraulic overload, which causes reductions in RV contractile performance [2,16].

In some reports on COVID-19 patients, marked RV dilation without signs of RV-pressure overload or pulmonary hypertension has also been described, suggesting that myocarditis is the causal mechanism of RV involvement [17]. Therefore, several pathophysiological mechanisms may be associated with RV dysfunction during infection with SARS-CoV2.

The RV-FWLS, based on the bi-dimensional speckle-tracking method, is a prognostic, reliable, and accurate tool for the evaluation of RV systolic function in cardiovascular diseases [18,19]. More recently, strain analysis has demonstrated to detect RV dysfunction in COVID-19 patients more sensitively and accurately than conventional echocardiographic RV functional parameters, such as TAPSE, tricuspid S’ or RVFAC [15]. Furthermore, RV-GLS was also shown to be a powerful predictor of greater mortality in patients with COVID-19 [5]. However, while the detection of the acute effect of COVID-19 pneumonia and PE on RV function by GLS has been extensively described, the data on the extent of myocardial damage following SARS-CoV2 infection are scarce. Bieber et al., in a cohort of 32 patients with COVID-19-associated myocardial injury, described a partial resolution of LV and RV dysfunction by GLS at two-months follow-up [20]. Luchian et al. reported, in patients with persistent dyspnea one year after COVID-19, the impairment of LV performance, demonstrated by a reduction in the LV GLS, myocardial work index, and constructive work, but not in RV [21]. Conversely, in a larger cohort of patients followed up for a mean of 129 days, an improvement in LV and RV longitudinal function was observed, mainly in those with impaired baseline function [22]. To the best of our knowledge, this is the first study to assess the impact of PE on myocardial performance at long-term follow-up after acute infection. In fact, we showed that one year after acute infection, in patients who experienced a thromboembolic event, RV subclinical dysfunction persisted and was detectable through significant reductions in RV-GLS and RV-FWLS. In our PE+ population, echocardiographic signs of pulmonary hypertension were not evident, despite a relevant, but not statistically significant, reduction in RVOT acceleration time. Moreover, a trend of increased tricuspid valve velocity and tricuspid regurgitation gradient was found, through the expression of increased pulmonary vascular resistance in the PE+ group compared to the patients without PE. Conversely, other parameters of RV dimension and function appeared to be comparable between the two groups, except for the strain values. A possible explanation could be that the usual parameters used to assess RV performance only represent a small portion of the RV, only address RV contraction in its longitudinal component, and are dependent on the angles of their measurements; therefore, they may be insensitive to subtle changes in RV function. Instead, RV strain, which is less angle- and load-dependent, and less influenced by passive tethering, allows the accurate quantification of regional and global myocardial function, reflecting myocardial contractility more closely. An accurate assessment of RV function is crucial in pathological conditions such as PE, in which the increase in RV afterload can no longer be accommodated by the RV contractile reserve, with the consequent occurrence of uncoupling [23]. At first, the uncoupling takes place during exercise or acute illness, but it then becomes detectable even at rest, in more advanced stages of the disease, when worsening dyspnea and systemic venous congestion signs occur. The detection of subclinical RV dysfunction may help to better understand the extent of myocardial damage in COVID-19 survivors, which may be responsible for the persistent dyspnea at mid- and long-term follow-up, and may provide powerful prognostic insights. Moreover, the evidence of RV-FWLS < 21% in recovered COVID-19 patients with suspected pneumonia might suggest a condition involving pulmonary pressure overload, such as undetected PE, which should be further explored with a CT scan.

The main limitations of the present study are its small sample size and monocentric character. However, our analysis was focused on COVID-19 patients with a confirmed diagnosis of PE, which is a known complication of COVID-19 disease, but that is not frequently diagnosed. Moreover, baseline echocardiographic data from the acute phase of COVID-19 disease were not available. Another limitation of the study is that at one-year follow-up, no functional respiratory evaluations were performed. Although the clinical and echocardiographic data were prospectively collected, our study was observational and retrospective; thus, it was subject to inherent flaws related to its design and the sample size may not have provided sufficient power to draw definite conclusions.

## 5. Conclusions

In recovered COVID-19 patients with a history of PE, there is the persistence of subclinical RV dysfunction one year after the acute phase of the disease, with values of RV-GLS and RV-FWLS that were significantly reduced compared to patients without PE. In this setting, a value of RV-FWLS lower than 21% was independently associated with PE. Thus, RV longitudinal strain can help to better analyze the long-term impact of COVID-19 on myocardial function, with potential important prognostic implications. However, larger prospective studies are needed to confirm our findings and definitely assess the role of RV-FWLS in predicting the occurrence of pulmonary embolism in COVID-19 population.

## Figures and Tables

**Table 1:** Baseline demographic, clinical, and biological characteristics.

Variables	Overall (n = 44)	PE+ (n = 22)	PE− (n = 22)	*p*-Value
Age, years	58.4 ± 13.3	58.7 ± 12.6	58 ± 14.3	0.859
Male, gender *n* (%)	31 (70)	16 (73)	15 (68)	0.741
Body-mass index, kg/m^2^	30.3 ± 6.3	30.9 ± 5.4	29.8 ± 7.1	0.572
Body surface area, m^2^	2.0 ± 0.2	2.0 ± 0.2	2.0 ± 0.3	0.891
Systolic blood pressure, mmHg	131.7 ± 15.1	130.36 ± 17.7	133.53 ± 11	0.523
Diastolic blood pressure, mmHg	78.9 ± 10.8	77.27 ± 11.72	81.18 ± 9.44	0.270
Obesity, *n* (%)	19 (43)	11 (50)	8 (36)	0.361
Hypertension, *n* (%)	29 (66)	16 (73)	13 (59)	0.340
Dyslipidemia, *n* (%)	14 (32)	9 (41)	5 (23)	0.195
Diabetes mellitus, *n* (%)	7 (16)	4 (18)	3 (14)	0.680
Smoking, *n* (%) - Non-smoker - Current smoker - Former smoker				0.834
	33 (75)	17 (77)	16 (73)	
	3 (7)	1 (4)	2 (9)	
	8 (18)	4 (18)	4 (18)	
Period of hospitalization, days	24.1 ± 12.5	30.2 ± 10.3	17.7 ± 11.6	0.001
Period of hospitalization in ICU, days	0.7 ± 3.1	1.3 ± 4.3	0	0.175
Mechanical ventilation, *n* (%)	2 (4)	2 (9)	0	0.175
Hemodynamic support, *n* (%)	2 (4)	2 (9)	0	0.175
Troponin I Hs, ng/mL	12.9 ± 41.6	19.2 ± 53.9	3.9 ± 5.1	0.412
D-dimer, μg/L	417 (200–2053)	620 (293–2898)	340 (88–1099)	0.025
C reactive protein, mg/L	11.3 ± 15.6	12.8 ± 19.8	9.5 ± 8.3	0.524
IL-6	187 (40–467)	279 (159–544)	50 (29–365)	0.017
PaO_2_/FiO_2_	163.9 ± 71.3	169.2 ± 78.5	150.6 ± 51.0	0.569
Lung total severity score	9.3 ± 3.8	10.8 ± 3.1	7.3 ± 3.9	0.004
Bilateral PE extension		9 (41%)		
No. of lobes affected by PE				
- 1		13 (59)		
- ≥2		9 (41)		

Values are *n* (%), mean ± SD or median (interquartile range). ICU = intensive care unit; IL = interleukin; PE = pulmonary embolism.

**Table 2:** Echocardiographic characteristics of the left chambers.

Variables	Overall (n = 44)	PE+ (n = 22)	PE− (n = 22)	*p*-Value
Interventricular septum, mm	10.5 ± 1.3	10.7 ± 1.12	10.3 ± 1.5	0.316
LV posterior wall, mm	9.9 ± 1.3	10 ± 1.36	9.73 ± 1.32	0.435
LV end-diastolic diameter, mm	46.5 ± 4.6	47.3 ± 4.3	45.6 ± 4.9	0.221
LV end-systolic diameter, mm	28.6 ± 5.0	29.9 ± 5.2	27.2 ± 4.6	0.113
LV mass indexed, g/m^2^	84.0 ± 20.9	87.6 ± 16.9	80.4 ± 24.2	0.263
Relative wall thickness	0.41 (0.38–0.46)	0.40 (0.37–0.48)	0.43 (0.41–0.46)	0.444
LV end-diastolic volume, mL	89.2 ± 26.1	87.2 ± 20.3	91.2 ± 31.3	0.614
LV end-systolic volume, mL	36.7 ± 13.0	36.4 ± 10.7	37.1 ± 15.2	0.864
LV ejection fraction, %	60.1 ± 5.3	58.9 ± 5.62	61.4 ± 4.9	0.129
LV GLS, %	19.6 ± 3.2	19.1 ± 3.0	20 ± 3.32	0.320
Indexed left atrial volume, mL/m^2^	22.5 (20.0–25.3)	23.5 (19.8–26.1)	21.7 (20.0–24.5)	0.647
Mitral E/A ratio, (m ± SD)	0.88± 0.23	0.85 ± 0.28	0.90 ± 0.18	0.440
Deceleration time, ms (m ± SD)	231.6 ± 55.3	236.2 ± 50.2	227.1 ± 60.9	0.590
E/e′, average (m ± SD)	8.0 ± 2.5	9.3 ± 2.5	7.0 ± 2.0	0.015

Values are mean ± SD or median (interquartile range). GLS = global longitudinal strain; LV = left ventricle.

**Table 3:** Echocardiographic characteristics of the right chambers.

Variables	Overall (n = 44)	PE+ (n = 22)	PE− (n = 22)	*p*-Value
Indexed right atrial volume, mL/m^2^	16.8 ± 4.5	15.8 ± 4.13	17.98 ± 4.8	0.117
RV basal diameter, mm	35.1 ± 4.9	36.3 ± 5.3	34 ± 4.2	0.126
RV FAC, %	48.8 (40.6–54.5)	50.6 (41.5–57.0)	47.7 (40.4–54.5)	0.390
TAPSE, mm	25.1 ± 4.1	25.5 ± 4.4	24.7 ± 3.9	0.565
RV S′, cm/s	13 (11–16)	13 (11–15)	12 (11–17)	0.989
Tricuspid valve velocity, m/s	2.3 (1.9–2.6)	2.4 (2.1–2.6)	1.9 (1.8–2.5)	0.089
Tricuspid regurgitation gradient, mmHg	22 (25–27)	23.5 (17.5–27.2)	15 (14–24)	0.062
sPAP, mmHg	30 (23–33)	32 (24–33)	27 (23–32)	0.467
TAPSE/sPAP	0.90 ± 0.25	0.85 ± 0.24	0.96 ± 0.26	0.308
RV GLS, %	19.0 ± 4.4	16.4 ± 2.9	21.6 ± 4.3	**<0.001**
RV-FWLS, %	21.8 ± 5.4	18.9 ± 4	24.6 ± 5.12	**<0.001**
RVOT acceleration time, msec	102.4 ± 23.3	95.9 ± 18.8	109.2 ± 26	0.074
Main pulmonary artery diameter, mm	21.9 ± 2.9	21.5 ± 2.9	22.3 ± 2.9	0.448

Values are mean ± SD or median (interquartile range). FAC = fractional area change; GLS = global longitudinal strain; RV = right ventricle; RV-FWLS = right-ventricle free wall longitudinal strain; RVOT = RV outflow tract; sPAP = systolic pulmonary artery pressure; TAPSE = tricuspid annular plane systolic excursion.

**Table 4:** Univariable and multivariable predictors of right-ventricle free wall strain below 21%.

Parameters	Univariate		Multivariate	
	HR (95% CI)	*p*-Value	HR (95% CI)	*p*-Value
Pulmonary thromboembolism	23.8 (3.990–141.963)	0.001	34.961 (3.241–377.091)	0.003
Obesity	4.444 (1.118–17.668)	0.034	10.341 (1.052–101.684)	0.045
Hypertension	7 (1.277–38.358)	0.025	-	-
RVOT acceleration time	0.970 (0.937–1.004)	0.08	-	-

CI = confidence interval; HR = hazard ratio; RVOT = right-ventricle-outflow tract.

## Data Availability

The data presented in this study are available on request from the corresponding author.

## Supplementary Materials

The following supporting information can be downloaded at: https://www.mdpi.com/article/10.3390/jcm12113611/s1, Table S1: Clinical characteristics at 1 year follow-up.
