# Supplementary material for: Effects on Right Ventricular Function One Year after COVID-19-Related Pulmonary Embolism
Source: J Clin Med. 2023 May 23;12(11):3611. doi: 10.3390/jcm12113611 (PMC10253748; doi:10.3390/jcm12113611)
Supplement: Supplementary file 1 [file jcm-12-03611-s001.zip › jcm-2347998-supplementary.pdf]

Supplementary Table S1. Clinical characteristics at 1 year follow-up.

Variables	Overall (n = 44)	PE+ (n = 22)	PE− (n = 22)	p-Value
**SpO_2**	96.0 ± 2.3	95.5 ± 2.3	96.4 ± 2.3	0.243
**NYHA class**				0.709
**I**	35 (80)	18 (82)	17 (77)	
**II**	9 (20)	4 (18)	5 (23)	
**≥ III**	0	0	0	

Values are n (%) or mean ± SD

NHYA = New York Heart Association; SpO_2 = peripheral oxygen saturation.
